# Feasibility of recruiting high-risk women in the US for HIV vaccine efficacy trials (HVTN 906)

**DOI:** 10.1186/1742-4690-9-S2-P123

**Published:** 2012-09-13

**Authors:** B Koblin, B Metch, C Morgan, R Novak, E Swann, D Metzger, D Lucy, D Dunbar, P Graham, T Madenwald, G Escamia, I Frank

**Affiliations:** 1 New York Blood Center, New York, NY, USA; 2 SCHARP / Fred Hutchinson Cancer Research Center, Seattle, WA, USA; 3 HIV Vaccine Trials Network / Fred Hutchinson Cancer Research Center, Seattle, WA, USA; 4 University of Illinois at Chicago, Chicago, IL, USA; 5 Division of AIDS, NIAID, NIH, Bethesda, MD, USA; 6 University of Pennsylvania, Philadelphia, PA, USA

## Background

Identifying women in the US with sufficient risk of HIV infection for inclusion in HIV vaccine efficacy trials has been challenging. Using geography and sexual network characteristics to inform new recruitment strategies, HVTN 906 determined the feasibility of recruiting and retaining women at high risk and assessed HIV incidence.

## Methods

HIV uninfected women were enrolled in Chicago, New York City and Philadelphia if they were 18-45 years, not pregnant or intending to become pregnant for 18 months and reported unprotected vaginal/anal sex in the prior six months and either i) resided or engaged in risk behavior in local geographical HIV risk pockets; and/or ii) had a male partner who had either been incarcerated or injected drugs in the last year or had concurrent sex with another partner in the last six months. Behavioral risk assessment, risk reduction counseling, HIV and pregnancy testing were done at baseline, 6, 12 and 18 months.

## Results

Among 799 women, 71% were from local high-risk pockets and had high-risk male partners, 18% were from local high-risk pockets only and 10% had high-risk male partners only. Median age was 37 years; 79% were Black and 15% Latina. At baseline, the median number of male partners was 3 (25%,75%: 2,7), 76% had unprotected sex while intoxicated (alcohol or drugs), and 52% exchanged sex for money or drugs. Retention at 18-months was 80%. Pregnancy incidence was 11% with 48% of pregnancies occurring during the first 6 months of follow-up. HIV incidence was 0.31% (95% CI: 0.06,0.91). Risk behaviors decreased between screening and 6 months with little change thereafter.

## Conclusion

Women recruited using new strategies based on geography and sexual network characteristics did not have a substantial HIV incidence, despite baseline levels of risk behaviors. New strategies to identify women at high risk in the US are needed.

